# In Memoriam: David Farrington, Founding Chair of the Campbell Crime and Justice Group

**DOI:** 10.1002/cl2.70045

**Published:** 2025-04-22

**Authors:** Lawrence W. Sherman, Friedrich Loesel, Dorothy Newbury‐Birch, David Weisburd

**Affiliations:** ^1^ Institute of Criminology University of Cambridge Cambridge UK; ^2^ University of Cambridge Cambridge UK; ^3^ Department of Law, Policing and Investigation, Cochair Campbell Crime & Justice Teeside University Middlesbrough UK; ^4^ George Mason University Fairfax Virginia USA; ^5^ Hebrew University Jerusalem Israel

In late 1997, the key founder of the medical Cochrane Collaboration, Ian Chalmers, called Larry Sherman from Oxford to discuss the recent “Maryland Report” (*Preventing Crime: What Works? What Doesn't? What's Promising?*) submitted to the US Congress by Attorney General Janet Reno and her Assistant Attorney General, Laurie Robinson. The report had attempted to undertake a series of semi‐systematic reviews in all areas for which federal funding had been made available. Sherman directed the project at the University of Maryland, where the co‐authors collaborated. All of them had been informed, in part, by the systematic reviews that David Farrington had already published at Cambridge, such as his 1981 review of randomized experiments in crime and justice.

Chalmers was not the only person to suggest that the Maryland report should become a springboard for an ongoing process of systematic reviews. The same idea struck a Philadelphia radio broadcaster, Jerry Lee, who contacted Sherman in mid‐1997 to discuss his possible donations to make that continuation happen. Within months, Jerry Lee had donated major funding to Maryland, including money for a visiting professorship at Maryland for Cambridge Professor David Farrington.

Farrington's engagement with the Maryland authors and Jerry Lee's support coincided with the developing support of the Cochrane community. By April 2000, Sherman delivered his inaugural lecture at the University of Pennsylvania, where by 2003, he founded its criminology department. The April 2000 event turned into a joint event for founding the Campbell Collaboration (named after program evaluation pioneer Donald T. Campbell) under the leadership of Penn Professor Robert Boruch (a former colleague of Campbell's and a renowned methodologist in social experiments).

A central actor in this effort was to be the first Chair of the Campbell Coordinating Committee on Crime and Justice, one of three initial substantive committees for overseeing systematic review production in social and human services. That Chair, by acclaim, was David P. Farrington. He was, for many reasons, an ideal choice. He had hundreds of co‐authors from all over the globe, including students and many others who had asked him to collaborate. He also had an incredibly broad range of subject matter interests, from juvenile delinquency to domestic abuse to crime prevention programs, from street lighting to cognitive therapies.

For the next 4 years, the Campbell Crime and Justice Group (CCJG) met up to twice yearly, with Farrington as the Chair and Jerry Lee's foundation supporting meetings in Europe—and in conjunction with annual meetings of the American Society of Criminology. Farrington's leadership attracted a group of outstanding scholars, all of whom with years of experience in conducting and reviewing evaluations of anticrime programs. Farrington led these meetings to highly productive results. He also ensured that peer reviewers with appropriate expertise were found to move the Campbell Reviews to completion. His commitment to publication also ensured high visibility and policy influence from the Committee's work.

The global character of the reviews was aided by his recruitment of not just US and UK citizens but also Asians and Europeans. One of the most active members was Germany's Stockholm Prize‐winning criminologist, Friedrich A. Loesel. As a leader of German criminology, Loesel collaborated with Farrington since the 1980s in the Advanced Research Center *Prevention and Intervention in Childhood and Adolescence* of the German Research Foundation. As first Presidents of the European Association of Psychology and Law, Loesel and Farrington promoted experimental research in this applied field of psychology. Both collaborated over many years in the CCJG and published together on program evaluations and protective factors against youth violence. Farrington and Loesel also collaborated on the Academy of Experimental Criminology, whose first President was Sherman in 1999. Farrington, as his successor, and several other presidents, produced a publication on the Academy's 20th Anniversary when Loesel was president. It summarized what had been achieved but also contained important perspectives for future experimental work and its relation to nonexperimental criminological research.

It is important to note David's commitment to the broader idea of advancing evidence‐based policy and experimental research. He played a key role in advancing the annual Jerry Lee Symposiums (supported by the Jerry Lee Foundation), which brought together major researchers, policymakers, and practitioners and showcased Campbell's efforts. During David's tenure as Chair, he encouraged David Weisburd to found the Journal of Experimental Criminology, published by Springer, which is now a top impact criminology journal.

David Farrington also benefited from the teams associated with David Weisburd, who, with collaborators, produced early reviews of such topics as problem‐oriented policing, community policing, second responders, and DNA testing. David F. also worked with David W. and Lawrence Sherman in developing funding from the National Institute of Justice and other sources, which provided critical support to the Jerry Lee Foundation for the activities of the CCJG. Farrington was not just a leader; he worked to mentor his younger colleagues to take leadership roles. David F. invited David W. to be Co‐Chair (2004) and then Chair of the Crime and Justice Group (from 2008).

Farrington and Weisburd worked to expand the recognition of the CCJG, for example, by authoring a Criminologist article on the group's efforts in 2007 (https://asc41.org/wp-content/uploads/ASC-Criminologist-2007-01.pdf). Earlier, in 2002, Farrington, along with Anthony Petrosino (who worked with David F. as an editor for the group in its early years) and Weisburd wrote an article for *The Forum*, a publication of the Justice Research and Statistics Association (19, no. 2: 1–6). During this period, Farrington and Weisburd placed great emphasis on extending the number of Systematic Reviews that could be published. This was greatly aided by the appointment of David Wilson as editor for the CCJG.

Farrington contributed to the CCJG a number of systematic reviews and meta‐analyses with colleagues, for example, on developmental crime prevention, programs against school bullying and offender treatment, effects of street lighting on crime, and parental imprisonment effects on children. His systematic reviews and meta‐analyses with Maria Ttofi and Hannah Gaffney on school‐based programs against bullying perpetration and victimization had particularly high view counts. In parts of this study, he also collaborated with Loesel, who became director of the Institute of Criminology at Cambridge (2005–2012). Since Farrington retired in 2012, he had been tireless in publishing from his world‐famous Cambridge Study in Delinquent Development, numerous other topics, and, in particular, on experiments in criminology. He had lit the torch of the CCJG and held it high until he passed away on November 5, 2024. For further information on his outstanding work, see the obituary for Farrington that Loesel wrote on request of the European Society of Criminology (Loesel 2024: https://escnewsletter.org/archive/in-memoriam-david-p-farrington).

